# Isolated del(5q) with myeloproliferative driver mutations: A systematic review of published cases and clinical implications

**DOI:** 10.1177/20406207261474937

**Published:** 2026-08-17

**Authors:** Mohammed Abdulgayoom, Abdulrahman F. Al-Mashdali, Awni Alshurafa, Mohammad S. Afana, Anas M. Babiker, Mohammad Bakheet, Shehab F. Mohamed, Mohamed A. Yassin

**Affiliations:** 1 Department of Hematology, Hamad Medical Corporation, 62850 National Center for Cancer Care and Research, Doha, Qatar; 2 College of Medicine, 225788 Omdurman Islamic University, Khartoum, Sudan

**Keywords:** myeloid neoplasms, del(5q), *JAK2 V617F*, *CALR*, *MPL*, MDS/MPN overlap

## Abstract

**Background:**

Myeloid neoplasms harboring both an isolated deletion of chromosome 5q (del(5q)) and myeloproliferative neoplasm (MPN) driver mutations (*JAK2, CALR, MPL*) constitute a rare and diagnostically challenging subset, often described with features overlapping those of myelodysplastic syndromes (MDS) and MPN.

**Methods:**

We systematically reviewed published case reports and case series of adult patients with myeloid neoplasms and concomitant isolated del(5q) and MPN driver mutations. A comprehensive search of PubMed and Google Scholar from inception through 31 December 2024 was performed using terms related to “del(5q)”, “*JAK2”, “CALR”, “MPL”*, and “myeloid neoplasm”. Eligible reports required documentation of isolated del(5q) and at least one MPN driver mutation with extractable clinical, cytogenetic, and/or molecular data. Data were synthesised descriptively; no quantitative meta-analysis was feasible. The review followed PRISMA 2020 guidelines and was registered post-study at INPLASY2025120046.

**Results:**

Twenty publications reporting 24 patients (diagnosed between 2006 and 2021) met the inclusion criteria. The median age was 70.5 years with a female predominance. Most patients presented with macrocytic anemia, thrombocytosis, and megakaryocytic dysplasia, frequently accompanied by marrow fibrosis. *JAK2 V617F* was the predominant mutation, whereas *CALR* and *MPL* were rarely described. Lenalidomide achieved hematologic responses in 14 of 17 evaluable patients and cytogenetic responses in 8 of 16; *JAK2 V617F* clearance occurred in 4 of 13. During a median follow-up of approximately two years, 5 of 24 patients progressed to acute myeloid leukemia.

**Conclusion:**

Reported cases with isolated del(5q) and an MPN driver mutation suggest a rare overlap presentation with mixed dysplastic and proliferative features. Lenalidomide appears to provide hematologic and cytogenetic benefit in some patients, although molecular persistence and progression to AML have been observed. Given the limited number and heterogeneity of published cases, these observations should be interpreted with caution. Larger, systematically collected datasets are needed to better understand the clinical significance and optimal management of this combination.

## Plain language summary

Chromosome 5q deletion [del(5q)] is a well-recognized abnormality in some bone marrow cancers called myelodysplastic syndromes (MDS). Patients with isolated del(5q) usually have anemia and respond well to the drug lenalidomide. In contrast, another group of blood cancers, called myeloproliferative neoplasms (MPN), are driven by specific mutations such as JAK2, CALR, or MPL, and often present with high platelet counts or bone marrow scarring. Although these two disease groups are usually separate, a small number of patients carry both an isolated del(5q) and an MPN-associated mutation. This unusual overlap creates a “grey zone” that is difficult to classify and manage, as the disease shows features of both MDS and MPN. In this study, we systematically reviewed all published case reports and case series describing such patients. We identified 24 patients reported between 2006 and 2021. Most were older adults, often women, who presented with anemia, high platelet counts, and abnormal bone marrow findings. The majority carried the JAK2 V617F mutation. Lenalidomide was given to most patients and led to improvements in blood counts in 14 out of 17 patients and disappearance of the chromosome abnormality in 8 out of 16 patients. However, the JAK2 mutation usually persisted, and 5 patients eventually developed acute myeloid leukemia, a more aggressive form of blood cancer. Our findings suggest that while lenalidomide is active in this overlap group, the disease remains at risk for progression, likely because the MPN clone is not fully eliminated. This highlights the importance of close monitoring and molecular testing. Because this condition is very rare, larger collaborative studies and registries are needed to clarify how best to classify and treat these patients.

## Article highlights

• Reported cases of del(5q) with MPN driver mutations suggest a diagnostic “grey zone” within myeloid neoplasms.
• Patients commonly present with macrocytic anemia, thrombocytosis, and megakaryocytic dysplasia, often with marrow fibrosis.
• Lenalidomide appears to induce frequent hematologic and cytogenetic responses, though molecular clearance of JAK2 is limited.
• Despite initial responses, progression to acute myeloid leukemia has been described in a subset of patients.
• The overlap phenotype likely reflects interplay between ribosomal haploinsufficiency and JAK–STAT pathway activation.
• Evidence is limited to case reports; prospective registries and integrated molecular profiling are needed to clarify classification and guide therapy.

## Introduction

Myeloid neoplasms encompass clinically and genetically distinct entities, most notably myelodysplastic syndromes (MDS) and myeloproliferative neoplasms (MPN).^1^ An isolated deletion of the long arm of chromosome 5 [del(5q)] defines a distinct MDS subtype characterized by macrocytic anemia, normal or elevated platelets, and megakaryocytic dysplasia, with well-recognized sensitivity to lenalidomide.^2,3^ By contrast, classical MPNs are driven by somatic mutations in *JAK2*, *CALR*, or *MPL*, leading to constitutive JAK–STAT signaling and proliferative hematopoiesis.^4–6^

Although typically associated with separate disease categories, these pathogenic drivers may occasionally coexist, creating a diagnostic “grey zone” that blurs the boundaries between MDS and MPN, which may be best considered within the spectrum of MDS/MPN overlap syndromes.^7–12^ Such dual-hit cases raise important biological and clinical questions: Does this combination represent a distinct overlap phenotype, a variant within existing classifications, or the result of convergent clonal evolution? What are the therapeutic implications—particularly regarding lenalidomide response—and how should prognosis be assessed?

Emerging molecular data suggest multiple possible models for the interaction between del(5q) lesions and MPN-driver mutations. In some cases, these alterations arise in independent or discordant clones, producing parallel MDS- and MPN-like populations with differential therapeutic sensitivities.^13,14^ In other reports, sequencing studies suggest a shared ancestral or same-clone origin, where del(5q)-associated ribosomal haploinsufficiency and p53 activation^15^ intersect with JAK–STAT–mediated proliferative signaling,^16^ producing a unified clone with mixed dysplastic–proliferative features. Experimental and clinical data showing that JAK2 V617F–mutant clones can expand through a competitive growth advantage provide a biological context for how *JAK2*-mutant and del(5q) clones might arise within the same patient, either sequentially or within related clonal lineages.^17^ Clinically, this may manifest as hybrid features, including macrocytic anemia, thrombocytosis, dysplastic megakaryocytes, and marrow fibrosis.

This systematic review synthesizes published cases of myeloid neoplasms harboring both isolated del(5q) and MPN driver mutations. We highlight their clinicopathological features, therapeutic responses—particularly to lenalidomide and risk of progression. Beyond evidence synthesis, we discuss the biological and clinical implications of this overlap and propose priorities for future research and disease classification.

## Methods

### Study design and search strategy

**Figure 1.**
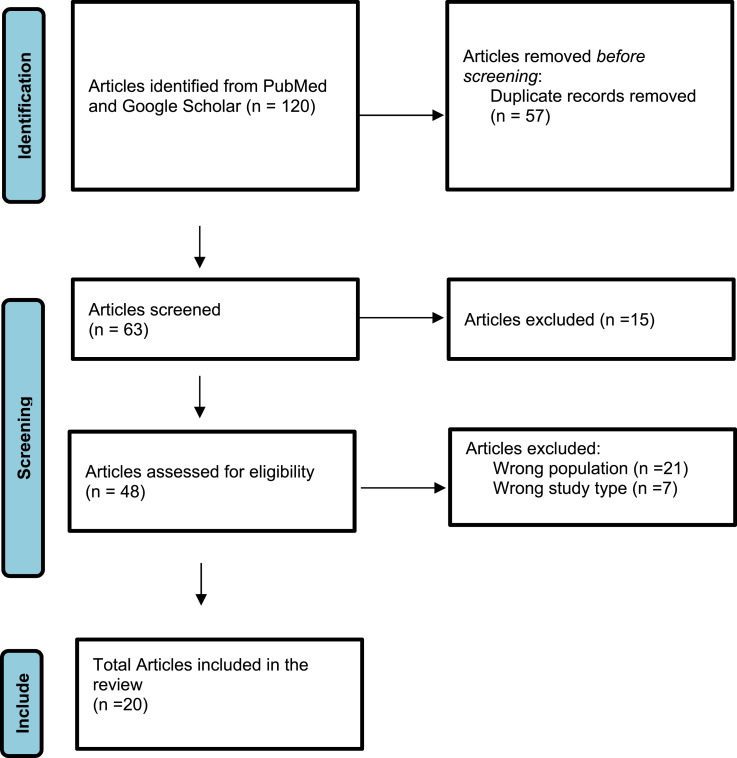
Flowchart of search strategy. PRISMA 2020 flow diagram illustrating the identification, screening, eligibility assessment, and inclusion of studies in this systematic review. A comprehensive literature search was performed in PubMed and Google Scholar from database inception through December 31, 2024. Search terms included combinations of: *“5q deletion,” “del(5q),” “JAK2,” “CALR,” “MPL,” “myeloid neoplasm,” “MDS,” “MPN,” “MDS/MPN,” “AML,” “case report,” and “case series.”* Only studies published in English and involving adults (≥18 years) were considered.

This study is a systematic review of published case reports and case series focusing on adult patients with myeloid neoplasms (including myelodysplastic syndromes [MDS], myeloproliferative neoplasms [MPN], and MDS/MPN overlap syndromes) harboring both an isolated 5q deletion and a driver mutation in *JAK2, CALR,* or *MPL*. The review was conducted in accordance with the Preferred Reporting Items for Systematic Reviews and Meta-Analyses (PRISMA) guidelines (Figure 1). The completed checklist is provided in Supplementary Appendix S1. The study protocol was registered post-study with INPLASY (registration number: INPLASY2025120046).

### Eligibility criteria

#### Inclusion criteria

1. Case reports or case series of adult patients (≥18 years) with a diagnosis of MDS, MPN, or MDS/MPN overlap.
2. Confirmed isolated 5q deletion (either alone or with one additional abnormality other than monosomy 7 or 7q deletion).
3. Documentation of at least one MPN driver mutation (JAK2, CALR, or MPL).
4. Sufficient clinical, cytogenetic, and/or molecular information available.
5. Reports labeled as AML were eligible only if an antecedent phase with isolated del(5q) plus an MPN driver mutation was clearly documented. In such cases, baseline features were extracted from the antecedent phase, and AML was captured as an outcome.

#### Exclusion criteria

1. Reports without adequate cytogenetic or molecular data.
2. Pediatric cases (<18 years).
3. Non-English publications.
4. De novo AML at presentation without a documented antecedent phase meeting inclusion criteria.

### Data extraction and handling of heterogeneity

Two reviewers independently extracted data using a standardized form; discrepancies were resolved by consensus or by a third reviewer. Extracted variables included demographics (age, sex), clinical features (symptoms, splenomegaly), laboratory data (Hb, MCV, platelet and WBC counts), bone marrow findings (cellularity, dysplasia, megakaryocyte morphology, fibrosis grade, ring sideroblasts, blast percentage), cytogenetic and molecular data (5q breakpoints, additional abnormalities, MPN driver mutation type and allele burden, co-mutations), and treatment/outcomes (hematologic, cytogenetic, molecular responses, progression to AML).

### Data synthesis and analysis

Findings were summarized using descriptive statistics (medians, ranges, and proportions). Given the heterogeneity of available reports and the case-report/series design, only qualitative synthesis was feasible. Where applicable, transformation to AML was analyzed as an outcome of antecedent disease.

## Results

### Search results

The systematic search identified 120 records. After removal of duplicates, 63 records were screened by title and abstract. Of these, 48 full-text articles were assessed for eligibility. Following exclusions (n = 21; reasons: Wrong population, data [n = 7], Wrong study type), 20 publications met inclusion criteria, yielding a total of 24 patients with coexisting isolated del(5q) and an MPN driver mutation (Figure 1).

### Patient and disease characteristics

Between 2006 and 2021, 20 publications describing 24 patients with dual genetic abnormalities were identified. The median age at diagnosis was 70.5 years (range, 49–85), with a female predominance (17 of 24). The diagnostic labels assigned by the original authors were heterogeneous: 8 patients were classified as MDS, 8 as MDS/MPN overlap syndromes, 5 as MPN, 1 as AML (with a documented antecedent phase), and 2 as unclassified myeloid neoplasms (Table 1).

**Table 1.** Baseline characteristics of patients with coexisting isolated del(5q) and MPN driver mutations. Baseline demographic and diagnostic characteristics of individual patients reported in published case reports and small case series describing coexisting isolated del(5q) and myeloproliferative neoplasm (MPN) driver mutations. Age, sex, and provisional diagnoses are presented as reported by the original authors. Diagnostic labels reflect historical classifications.

Study no.	Author, Year	Age (years)	Sex	Provisional diagnosis
1A	Bürki et al., 2018^18^	83	Female	MDS/MPN
1B	Bürki et al., 2018^18^	82	Female	MDS/MPN
2	Tremblay-LeMa et al., 2018^19^	68	Female	Not specified
3	Pich et al., 2016^20^	63	Female	MDS
4	Oka et al.,2017^21^	73	Female	MDS
5	Sokol et al., 2010^22^	56	Female	MDS
6	Nomdedeu et al., 2011^23^	54	Female	MDS
7	Azaceta et al., 2010^24^	85	Female	MDS
8	Alshaban et al., 2018^25^	80	Male	MDS/MPN-RS-T
9	Kirito et al., 2020^26^	70	Male	ET
10	Hatzimichael et al., 2016^27^	77	Female	MDS
11	Musto et al., 2014^28^	84	Male	MDS
12	Wong et al., 2010^29^	66	Male	ET
13	Dasanu et al., 2011^30^	73	Female	del(5q)-positive myeloproliferative neoplasm
14	Marcé et al., 2021^31^	82	Female	MDS/MPN
15A	Miotke et al., 2021^32^	49	Female	MDS/MPN
15B	Miotke et al., 2021^32^	72	Female	MDS/MPN
16	Vaccarino et al., 2016^33^	71	Female	MDS
17	Čolović N et al., 2019^34^	58	Male	MDS/MPN
18A	Tefferi et al., 2007^35^	75	Male	Post-PV MF
18B	Tefferi et al., 2007^35^	69	Female	Post-PV MF
18C	Tefferi et al., 2007^35^	63	Male	PMF
19	Kruszewski et al., 2016^36^	59	Female	RARS-T
20	Mesa et al., 2006^37^	82	Female	AML

Diagnostic assignments reflect those reported by original authors. Abbreviations: MDS/MPN-RS-T, MDS/MPN with ring sideroblasts and thrombocytosis; ET, essential thrombocythemia; Post-PV MF, post-polycythemia vera myelofibrosis; PMF, primary myelofibrosis; RARS-T, refractory anemia with ring sideroblasts and thrombocytosis (now reclassified as MDS/MPN-RS-T).

Clinically, most patients presented with macrocytic anemia, with a median hemoglobin of 9.6 g/dL (IQR, 7.7–10.0) and a median mean corpuscular volume of 106 fL (IQR, 103.8–111.8). Thrombocytosis was common, with a median platelet count of 737 ×10^3^/µL (IQR, 616–938). The median white blood cell count was 7.4 ×10^3^/µL (IQR, 5.6–14.2). Splenomegaly was reported in 7 of 18 evaluable cases. Presenting symptoms were largely anemia-related, although a minority reported B symptoms or pruritus.

### Bone marrow and genetic findings

Bone marrow examinations generally revealed hypercellularity with marked megakaryocytic hyperplasia and dysplasia, particularly hypolobulated small megakaryocytes (Table 2). Fibrosis was described in 10 of 14 evaluable patients, most often of moderate severity (Grade 2). Increased blasts (10–30%) were observed in 2 patients, one of whom also carried additional co-mutations (*DNMT3A, ASXL1, KRAS*, and *GATA2*).

**Table 2.** Comprehensive bone marrow findings. Summary of bone marrow morphologic features reported in published cases of patients with coexisting isolated del(5q) and MPN driver mutations. Denominators reflect the number of patients for whom each parameter was reported.

Category	Subcategory	Total patients	Findings	Notes
Overall Cellularity	–	23	Hypercellular: 21, Normocellular: 1, Hypocellular: 1	Predominantly hypercellular
Erythroid Lineage	Cellularity	13	Hyperplasia: 5, Normal: 3, Hypoplasia: 5	Mixed pattern
	Dysplasia	11	Present: 8, Absent: 3	72.7% dysplasia rate
Granulocyte Lineage	Cellularity	13	Hyperplasia: 6, Normal: 4, Hypoplasia: 3	–
	Dysplasia	11	Present: 8, Absent: 3	72.7% dysplasia rate
Megakaryocyte Lineage	Cellularity	17	Hyperplasia: 16, Hypoplasia: 1	Near-universal hyperplasia
	Dysplasia	20	Present: 20, Absent: 0	100% dysplasia rate
	Size (n=13)	13	Small: 8, Large: 3, Normal: 1, Mixed: 1	Small megakaryocytes dominate
	Nuclear lobulation	18	Present: 16, Hyperlobulated: 1, Mixed: 1	Hypolobulation is prominent
Bone Marrow Fibrosis	–	14	Present: 10 (Grade 1: 3, Grade 2: 5, Grade 3: 2), Absent: 4	Mostly moderate (Grade 2) fibrosis
Ringed Sideroblasts	–	10	Present: 2, Absent: 8	Rare

Cytogenetically, all patients harbored an isolated del(5q), most frequently spanning q13–q33. *JAK2 V617F* was the predominant mutation (22 of 24 cases), whereas *CALR* and *MPL* mutations were each reported only once. The *JAK2* variant allele frequency ranged from 2.7% to 80%, indicating marked clonal heterogeneity.

### Treatment and outcomes

**Figure 2.**
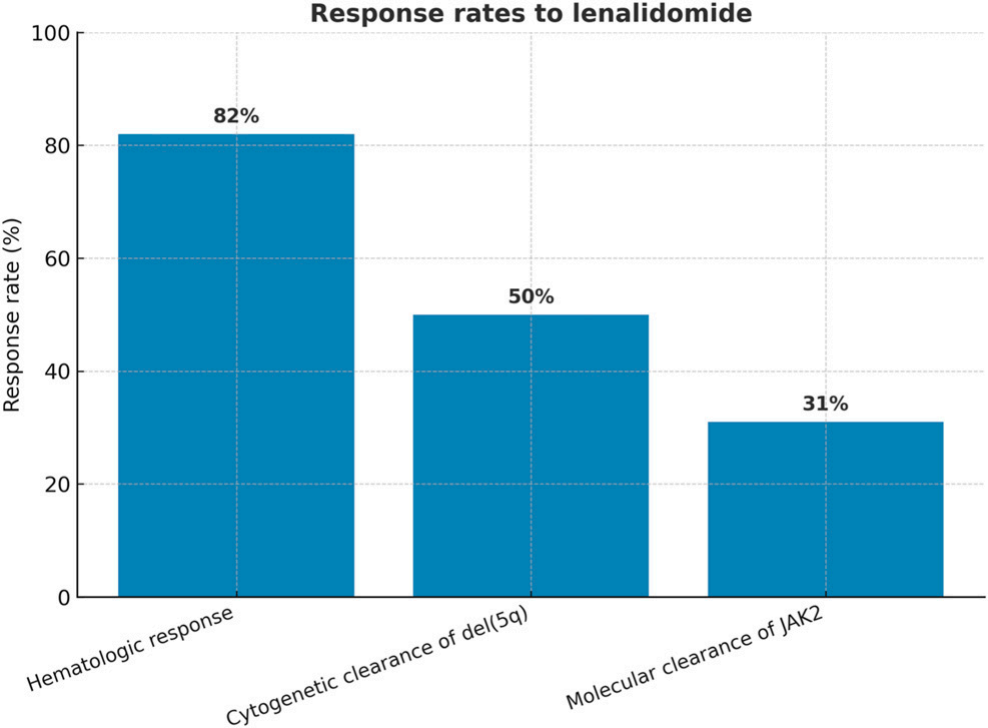
Response rates to lenalidomide in patients with del(5q) and MPN driver mutations. Distribution of hematologic and cytogenetic response categories to lenalidomide among reported patients with coexisting isolated del(5q) and myeloproliferative neoplasm (MPN) driver mutations. Response rates are calculated from available reported cases only, with denominators varying according to response type and completeness of reporting in the original publications.

Seventeen patients received lenalidomide, with encouraging responses reported. Hematologic improvement was observed in 14 of 17 evaluable patients (82%), while cytogenetic clearance of del(5q) occurred in 8 of 16 (50%). Molecular clearance of JAK2 V617F was achieved in 4 of 13 evaluable patients (31%) (Figure 2). Lenalidomide was generally well tolerated, with only one discontinuation due to pancytopenia.

In addition to lenalidomide, a subset of patients received conventional MPN-directed cytoreductive therapy. Hydroxyurea use was documented in at least seven cases, either prior to or contemporaneous with the diagnosis of del(5q). Interferon-α was reported in two patients, and one patient received anagrelide. However, for the majority of cases (approximately two-thirds), no cytoreductive therapy was used or reported, and information regarding treatment timing, dosing, and duration was inconsistently provided. Consequently, the available evidence was insufficient to determine whether prior or concomitant cytoreduction influenced hematologic or molecular response patterns or risk of progression.

During a median follow-up of approximately two years, 5 of 24 patients (21%) progressed to AML. Progressive marrow fibrosis was also reported in several cases. Adverse outcomes were more frequent among patients with additional co-mutations or a higher JAK2 allele burden.

## Discussion

### Diagnostic ambiguity and overlap biology

The coexistence of isolated del(5q) and MPN driver mutations represents a biologically and clinically ambiguous entity that challenges current WHO/ICC classifications.^1,7^ While classical del(5q) MDS is characterized by ineffective erythropoiesis and sensitivity to lenalidomide,^2,3,22–24^ the presence of JAK2, CALR, or MPL mutations introduces proliferative features such as thrombocytosis, splenomegaly, and marrow fibrosis.^5,6,18–20,26^ Nearly all cases in this review demonstrated this mixed phenotype, supporting the concept of a potential MDS/MPN overlap entity. Emerging molecular data provide possible explanations for this biology. Some reports describe independent or discordant clones, where dysplastic and proliferative populations coexist and exhibit differential treatment responses.^13^ In other patients, clinical and morphological features are more consistent with a shared ancestral clone, in which del(5q)-associated ribosomal haploinsufficiency and JAK–STAT activation converge within a single lineage. The competitive fitness and expansion capacity of JAK2 V617F demonstrated experimentally^17^ further support the predominance of proliferative traits observed in several cases. These frameworks mirror recent morphologic and population-based publications suggesting that del(5q) with MPN-driver mutations constitutes a reproducible overlap phenotype distinct from classical MDS or MPN categories.^12,14^

### Therapeutic implications of lenalidomide sensitivity

A notable finding was the high hematologic response rate, documented in 14 of 17 evaluable patients, and cytogenetic clearance of del(5q) in 8 of 16, resembling outcomes in classical del(5q) MDS.^2,3,22–25^ This pattern may indicate that del(5q)-associated pathways play a major role in lenalidomide sensitivity, but further confirmation is needed. However, molecular remissions of *JAK2* were relatively uncommon, observed in about 4 of 13 patients, indicating that the MPN clone often persists despite hematologic recovery.^18–20,22,23,27^ This partial clonal targeting may help explain the continued risk of progression—including transformation to AML—observed in 5 of 24 patients. Mechanistically, del(5q)-mediated ribosomal haploinsufficiency may sensitize to CK1α degradation,^15^ while *JAK2* activation could modulate p53 activity,^16^ together creating a paradoxical therapeutic vulnerability. These findings are hypothesis-generating and suggest a possible role for dual-targeting strategies, such as combining lenalidomide with *JAK–STAT* inhibitors, although this concept remains speculative and warrants evaluation in larger cohorts.

### Prognosis and risk of progression

Despite initial responses, outcomes were not uniformly favorable. Transformation to AML occurred in 5 of 24 patients, often within three years,^20,21,30,37^ and fibrotic progression was also reported.^18,19,25,35^ This appears more aggressive than the generally indolent course of classical del(5q) syndrome,^3,4^ suggesting—but not proving—that the presence of MPN drivers may confer a higher-risk trajectory. Additional somatic mutations, such as *ASXL1* and *DNMT3A,* may further influence prognosis,^8,9^ although available case-level data were insufficient to determine their precise impact. These findings underscore the importance of comprehensive molecular profiling at baseline and during follow-up to anticipate clonal evolution.

### Comparative context

To our knowledge, this review is the first to synthesize case-level data specifically on myeloid neoplasms with isolated del(5q) and MPN driver mutations. However, four retrospective cohorts have provided complementary insights into this rare phenotype (Supplementary Table S1).

The earliest recognition came from Ingram et al. (2006),^38^ who reported JAK2 V617F in 6 of 97 patients with del(5q) MDS, consistently associated with thrombocytosis and megakaryocytic hyperplasia. Wong et al. (2009)^39^ subsequently described three JAK2-positive del(5q) patients in a Chinese cohort, all of whom achieved robust hematologic responses to lenalidomide and showed a low risk of leukemic transformation, underscoring preserved drug sensitivity in this subgroup. By contrast, Patnaik et al. (2010)^40^ observed inferior outcomes in JAK2-positive patients (6.8% of 88 del(5q) cases), with shorter response durations (median 18 vs. 32 months) and reduced overall survival (hazard ratio 2.1), raising concerns about adverse prognostic impact. Finally, Sangiorgio et al. (2020)^41^ highlighted progression risks, reporting 6 JAK2-positive cases among 47 del(5q) patients, with 70% developing marrow fibrosis and 20% progressing to AML, indicating a potential predisposition to clonal evolution.

Nevertheless, outcomes varied between cohorts. Ingram et al. (2006)^38^ and Wong et al. (2009)^39^ reported preserved lenalidomide sensitivity and low risk of leukemic transformation, whereas Patnaik et al. (2010)^40^ observed inferior overall survival and shorter response durations in *JAK2*-positive patients. Sangiorgio et al. (2020)^41^ highlighted an AML progression risk of 5 of 24 patients, particularly in cases with marrow fibrosis. These discrepancies may reflect small sample sizes, patient selection differences, and variable follow-up, but they also suggest biological heterogeneity within this dual-hit group.

Collectively, these retrospective cohorts suggest a reproducible proliferative phenotype in del(5q) cases with MPN driver mutations, most notably thrombocytosis, megakaryocytic hyperplasia, and fibrosis—while also illustrating variability in clinical outcomes. Reported discrepancies across studies (e.g., 0% vs. 20% AML progression)^38–41^ may be explained by small sample sizes, differences in follow-up duration, or underlying clonal heterogeneity. Our case-level synthesis adds nuance to these observations by showing that, despite frequent hematologic and cytogenetic responses to lenalidomide, progression events—including transformation to AML in 5 of 24 patients—were still observed, underscoring the need for careful long-term monitoring. Together, these findings raise the possibility that del(5q) + MPN driver neoplasms represent a biologically distinct niche where ribosomal haploinsufficiency and JAK–STAT pathway activation coexist, but further prospective studies are needed to clarify their classification and prognostic relevance.

### Limitations

This synthesis is limited by the rarity of reported cases, the retrospective nature of the available evidence, and substantial heterogeneity in study design and reporting. Most data are derived from isolated case reports or very small case series, which limits generalizability and precludes formal meta-analysis. Although a systematic search was performed using PubMed and Google Scholar with manual reference screening, not all bibliographic databases were searched, and relevant reports may therefore have been missed. Publication bias is also likely, with preferential reporting of clinically notable or favorable outcomes. Molecular and clonal monitoring was inconsistently reported, limiting insights into clonal architecture and disease evolution. In addition, the absence of prospectively and systematically collected data precludes firm conclusions regarding survival, treatment efficacy, or comparative outcomes. Finally, the protocol was registered post-study, which may have introduced bias and represents an additional methodological limitation of this review.

## Conclusion

The coexistence of isolated del(5q) and MPN driver mutations remains a rare and incompletely defined entity. While lenalidomide demonstrates encouraging activity, progression to AML or fibrosis can still occur. Given the small number and heterogeneity of available reports, our conclusions are descriptive and hypothesis-generating. Prospective registries and collaborative studies are needed to better define disease characteristics, prognosis, and therapeutic strategies.

## Supplemental material

Supplemental material - Isolated del(5q) with myeloproliferative driver mutations: A systematic review of published cases and clinical implications Supplemental material for Isolated del(5q) with myeloproliferative driver mutations: A systematic review of published cases and clinical implications by Mohammed Abdulgayoom, Abdulrahman F. Al-Mashdali, Awni Alshurafa, Mohammad S. Afana, Anas M. Babiker, Mohammad Bakheet, Shehab F. Mohamed, and Mohamed A. Yassin in Therapeutic Advances in Hematology.

Supplemental material - Isolated del(5q) with myeloproliferative driver mutations: A systematic review of published cases and clinical implications Supplemental material for Isolated del(5q) with myeloproliferative driver mutations: A systematic review of published cases and clinical implications by Mohammed Abdulgayoom, Abdulrahman F. Al-Mashdali, Awni Alshurafa, Mohammad S. Afana, Anas M. Babiker, Mohammad Bakheet, Shehab F. Mohamed, and Mohamed A. Yassin in Therapeutic Advances in Hematology.

## Data Availability

This review is based on data extracted from previously published case reports, case series, and cohort studies, all of which are cited in the manuscript. Additional details are available from the corresponding author upon reasonable request.*
