# Supplementary material for: Isolated del(5q) with myeloproliferative driver mutations: A systematic review of published cases and clinical implications
Source: Ther Adv Hematol. 2026 Aug 17;17:20406207261474937. doi: 10.1177/20406207261474937 (PMC13487117; doi:10.1177/20406207261474937)
Supplement: Supplementary file 1 [file sj-pdf-1-tah-10.1177_20406207261474937.pdf]

Supplementary Appendix S1. PRISMA 2020 Checklist

Title of Review: *Isolated del(5q) with Myeloproliferative Driver Mutations: A Systematic Review of Published Cases and Clinical Implications*
Authors: Mohammed Abdulgayoom et al.
Registration: The study protocol was registered post-study with INPLASY (registration number: INPLASY2025120046)

PRISMA 2020 Checklist for Systematic Reviews

Section & Topic	PRISMA Item	Checklist Item Description	Location in Manuscript
**TITLE**	1	Identify the report as a systematic review.	Title page; Abstract, Methods
**ABSTRACT**	2	Structured summary including background, objectives, data sources, eligibility criteria, participants, interventions, study appraisal, synthesis methods, results, limitations, conclusions, and registration.	Structured Abstract
**INTRODUCTION**	3	Rationale for the review based on existing knowledge.	Introduction
	4	State the objectives, including research questions (PICO if applicable).	Introduction (final paragraph)
**METHODS**	5	Indicate if the review was registered, name the registry, and give the registration number.	Methods – Study Design and Search Strategy
	6	Present full eligibility criteria.	Methods – Eligibility Criteria
	7	All information sources (databases, coverage dates, search date).	Methods – Study Design and Search Strategy
	8	Provide full electronic search strategy for at least one database.	Methods – Search Strategy (and Supplement if added)
	9	State the process for selecting studies.	Methods – Study Selection
	10	Describe the methods of data extraction.	Methods – Data Extraction and Handling of Heterogeneity

	11	List all variables for which data were sought.	Methods – Data Extraction (variables listed)
	12	Methods for assessing risk of bias of included studies.	Not applicable (case reports/series; noted in Limitations)
	13	Describe the effect measures used.	Not applicable (qualitative synthesis only)
	14	Synthesis methods, approaches to heterogeneity, rationale for not performing meta-analysis.	Methods – Data Synthesis and Analysis
	15	Describe any methods used to assess certainty of evidence.	Not applicable (case-level descriptive data)
**RESULTS**	16	Study selection numbers, reasons for exclusions, and PRISMA flow diagram.	Results – Search Results; Figure 1
	17	Characteristics included studies.	Results – Patient and Disease Characteristics; Table 1
	18	Risk of bias for each study.	Not applicable (case reports/series)
	19	Results of individual studies.	Results – Tables 1 and 2
	20	Results of syntheses, including summary findings.	Results – Treatment and Outcomes
	21	Reporting biases assessment.	Limitations
	22	Certainty of evidence assessment.	Not applicable
**DISCUSSION**	23	Summary and interpretation of results.	Discussion
	24	Limitations of evidence and review processes.	Discussion – Limitations
	25	Implications for practice, policy, and future research.	Discussion & Conclusion
**OTHER INFORMATION**	26	Registration information.	Methods – Study Design and Search Strategy; Abstract
	27	Protocol availability.	INPLASY (registration number: INPLASY2025120046)

	28	Support/funding of the review.	Declarations – Funding
	29	Competing interests.	Declarations – Conflict of Interest Statement
	30	Availability of data, materials, and supplementary content.	Declara
