# Supplementary material for: Isolated del(5q) with myeloproliferative driver mutations: A systematic review of published cases and clinical implications
Source: Ther Adv Hematol. 2026 Aug 17;17:20406207261474937. doi: 10.1177/20406207261474937 (PMC13487117; doi:10.1177/20406207261474937)
Supplement: Supplementary file 2 [file sj-pdf-2-tah-10.1177_20406207261474937.pdf]

Supplementary table 1 . Retrospective Cohorts of del(5q) Myeloid Neoplasms with MPN Driver Mutations. Summary of retrospective cohort studies reporting the frequency, clinical features, and outcomes of myeloid neoplasms with isolated del(5q) harboring myeloproliferative neoplasm (MPN) driver mutations. Cohort size is shown as the number of del(5q) cases with MPN driver mutations relative to the total del(5q) population studied. Key findings and clinical implications are presented as reported by the original authors**.**

**Study**	**Cohort Size (del[5q]+MPN driver / total del[5q])**	**Key Findings**	**Clinical Implications**
Ingram et al. (2006) [34]	6/97 (6%) JAK2	Thrombocytosis, megakaryocytic hyperplasia	Suggests JAK2+ defines proliferative del(5q) subset
Wong et al. (2009) [35]	3/10 (30%) JAK2	100% erythroid improvement with lenalidomide, no AML progression	Preserved lenalidomide sensitivity
Patnaik et al. (2010) [36]	9/190 (4.7%) (5 JAK2, 3 MPL, 1 JAK2+MPL)	JAK2+ associated with shorter response (18 vs 32 mo) and worse OS (HR 2.1, p=0.04)	JAK2 predicts inferior survival
Sangiorgio et al. (2020) [37]	6/47 (13%) JAK2	70% fibrosis, 20% AML progression	Monitor for fibrotic/AML progression
